# Fabrication of gold-coated PDMS surfaces with arrayed triangular micro/nanopyramids for use as SERS substrates

**DOI:** 10.3762/bjnano.8.227

**Published:** 2017-11-01

**Authors:** Jingran Zhang, Yongda Yan, Peng Miao, Jianxiong Cai

**Affiliations:** 1 The State Key Laboratory of Robotics and Systems, Robotics Institute, Harbin Institute of Technology, Harbin, Heilongjiang 150080, P.R. China; 2 Center for Precision Engineering, Harbin Institute of Technology, Harbin, Heilongjiang 150001, P.R. China; 3 School of Chemistry and Chemical Engineering, Harbin Institute of Technology, Harbin, Heilongjiang 150001, P.R. China

**Keywords:** micro/nanopyramid, nanoimprinting, PDMS substrate, rhodamine 6G, SERS

## Abstract

Using the tip-based continuous indentation process, arrays of three-dimensional pyramidal cavities have been successfully machined on a copper template and the structures were successfully transferred to a polydimethylsiloxane (PDMS) surface using a reverse nanoimprinting approach. The structured PDMS surface is coated with a thin Au film, and the final substrate is demonstrated as a surface-enhanced Raman spectroscopy (SERS) substrate. Rhodamine 6G (R6G) was used as a probe molecule in the present study to confirm the SERS measurements. Arrays of micro/nanostructures of different dimensions were formed by the overlap of pyramidal cavities with different adjacent distances using the tip-based continuous indentation process. The effects of the reverse nanoimprinting process and coating process on the final topography of the structures are studied. The experimental results show that the Raman intensity of the Au-film-coated PDMS substrate is influenced by the topography of the micro/nanostructures and by the thickness of the Au film. The Raman intensity of 1362 cm^−1^ R6G peak on the structured Au-film-coated PDMS substrate is about 8 times higher than the SERS tests on a commercial substrate (Q-SERS). A SERS enhancement factor ranging from 7.5 × 10^5^ to 6 × 10^6^ was achieved using the structured Au-film-coated PDMS surface, and it was demonstrated that the method proposed in this paper is reliable, replicable, homogeneous and low-cost for the fabrication of SERS substrates.

## Introduction

Surface enhanced Raman spectroscopy (SERS) is a prominent, highly analytical tool for the detection of chemical molecules and biological species at low concentrations. SERS has recently attracted wide-ranging attention from researchers in many fields, including biosensing and bioanalysis [1–3], cancer cell work [4], virus identification [5–6], food safety [7–8] and gas vapor research [9–10]. As compared to traditional Raman scattering technology, SERS can provide significant signal amplification for analyte molecules absorbed on a SERS-active substrate and can extend the scope of Raman scattering to detect molecules at very low concentrations. Metals such as gold, silver and copper can be selectively employed to obtain surface enhancement with well-dispersed micro/nanostructures that provide the SERS sensor with an effective identification capability for adsorbed markers.

Typically, SERS substrates have been fabricated using lithography-based technologies [11–16], such as electron-beam lithography (EBL) [11–12], soft interference lithography (SIL) [13–14], and nanosphere lithography (NSL) [15–16]. To improve the reproducibility and production quantity of SERS substrates, researchers have focused on replicating molded micro/nanostructures as SERS substrates using imprint lithography. Several researchers have used biological organisms as biotemplates, such as the wings of cicadas [17–18] and butterflies [19–21]. A nanostructured SERS substrate was achieved for the replication of a biotemplate of a cicada wing and low concentrations of thiophenol and rhodamine 6G (R6G) were detected as test analytes [17]. The micro/nanostructures of a blue butterfly wing were used as a template, and a SERS substrate was produced and utilized to detect rhodamine dye for the elimination of organic pollutants [19]. Additionally, pyramidal array structures on conventional Klarite substrates [22] were fabricated as specifically engineered structures with an apex angle of 70.5° that can also be used as a template. The SERS substrate can then be achieved via a ultraviolet (UV) embossing process by transferring these structures to a plastic substrate. Templates [23–25] were also produced by a lithography-based method and reproducible plastic substrates were machined using different nanoimprinting methods [26]. For example, Courvoisier et al. [4] designed and fabricated an accurate inverted array of squares as a template on a silicon wafer via EBL and wet etching approaches. They produced arrays of tipless pyramids using an optical UV curing method. Lee et al. [24] used anodic aluminum oxide as a template for transferring patterns onto the polydimethylsiloxane (PDMS) substrate surfaces using a dry etching method. In this work, the detection of DNA molecules showed a selective enhancement of Raman scattering. Owing to good flexibility and excellent optical transparency, polymer substrates can come into direct contact with a sample surface of arbitrary shape. This is a task that is difficult to achieve for the SERS substrates with hard templates, including silicon and glass. A further advantage is that laser light can pass through the polymer substrate and reach the nanoparticle layer to activate the plasmon resonance, which generates the enormous SERS enhancement. A SERS substrate with good transparency and flexibility was prepared as a self-assembly of gold nanoparticles on a poly(methyl methacrylate) (PMMA) template, and malachite green on fish skin [27] was successfully detected. A flexible and transparent substrate consisting of silver nanoparticles on polyethylene terephthalate (PET) sheets was fabricated for in situ detection of R6G and thiram residues with a concentration of 10^−4^ mol on the skin of an apple and a cherry tomato, respectively [28]. The major limitations of these methods are in the complexity of the fabrication processes for obtaining the templates. Additionally, more complex micro/nanostructures are difficult to achieve using the existing methods.

Recently, the micro/nanomechanical machining method [29–31] has been employed to machine SERS substrates. The main characteristics of this method are that the depth and width of the micro/nanostructures can be easily and precisely controlled [32]. Commercial nanoindenters are mainly used to detect mechanical properties such as hardness, elastic modulus and friction coefficient. The fabrication of micro/nanostructures using the nanoindenter method can be also achieved. The shortcomings of this technique include low machining speed, small machining area and high cost, and thus the nanoindentation method is not suitable for mass production. Similar to traditional nanoindenter methods, in our previous study, a force modulation indentation method was also presented [33–34]. The force modulation indention equipment that we built improved the machining efficiency. By controlling the period and amplitude of the force signal in the vertical direction and the machining velocity of the precision stage in the horizontal direction simultaneously, arrayed micro/nanocavities were formed by the overlap of the pile-ups with high efficiency.

In the present study, arrays of triangular microcavities on a Cu(110) surface were fabricated by the force modulation indention method previously developed [33–34]. The method of nanoimprinting was employed to transfer these patterns (micro/nanostructures) from the Cu(110) substrate to the PDMS surface and the template can be reused for many times in the imprinting process. The Raman intensities of R6G were detected on the gold-coated, structured PDMS surface with different dimensions of micro/nanostructures. The effect of the gold film thickness on the PDMS substrate was studied as well. Finally, the Raman intensities of R6G on the pyramidal structures fabricated by this method were compared to a commercial substrate (Q-SERS). This method was verified to be a high-resolution, highly reproducible, and low cost approach to the fabrication of high-performance SERS substrates that could be used as sensors to detect pesticide residues on the skin of fruit or fish.

## Results and Discussion

### Fabrication of micro/nanopyramids on PDMS surfaces

**Figure 1:**
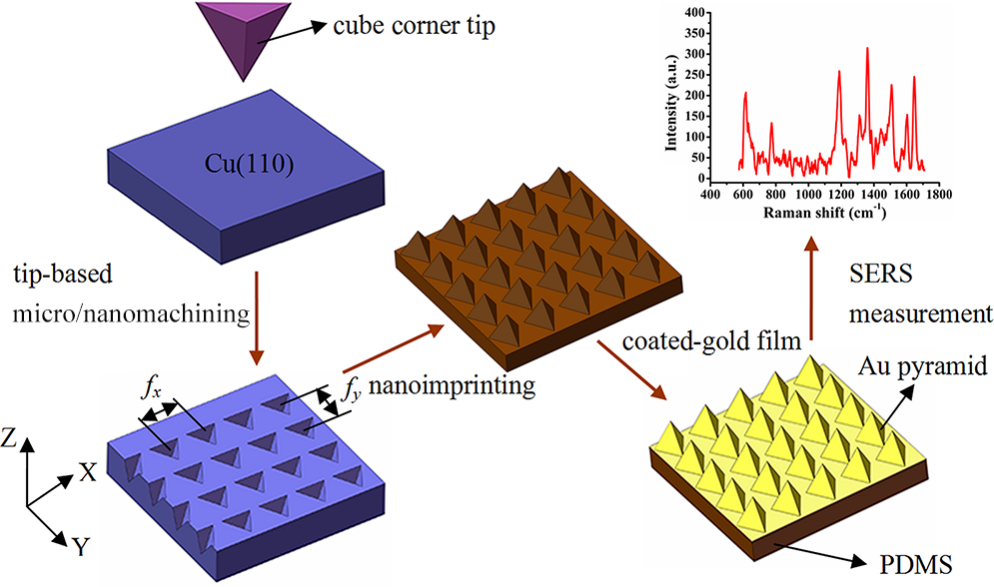
Schematic of the SERS substrate basic fabrication process steps with close-packed, arrayed Au pyramids.

Close-packed Au pyramidal arrays were fabricated using the inverted pyramidal pits on PDMS to form homogeneous and highly reproducible SERS substrates. The basic fabrication process steps are shown in Figure 1. First, a home-built tip-based force-modulated indentation system previously developed was used to fabricate periodic micro/nanocavities on the Cu(110) surface (Heifei Ke Jing Materials Technology Co., Ltd., China) using the cube corner tip. The distance between adjacent cavities was controlled by the feed, *f_x_* and *f_y_*, in the *X* and *Y* directions, respectively. The details of this method can be found in [30]. Table 1 lists the parameters of the feed trials and the fabrication time in the *X*–*Y* direction for an area of 30 × 30 μm^2^. Second, the PDMS base (Sylgard184A, Dow Corning) and hardener (Sylgard184B, Dow Corning) were mixed at a ratio of 10:1 in a petri dish. The Cu(110) substrate and PDMS were then heated at 80 °C for 2.5 h on a hot plate. After cooling, the fabricated structures on the Cu(110) surface were transferred to the PDMS substrate at an appropriate temperature using the reverse nanoimprinting approach. Finally, a gold film was deposited on the structured PDMS surface by electronic beam evaporation. The arrayed Au pyramids were formed as an active substrate that was used for the following Raman measurements.

**Table 1:** Parameters of the feed trials and fabrication time in the *X*–*Y* directions for machining the arrayed micro/nano structures on the template surface.

	(1)	(2)	(3)	(4)	(5)	(6)	(7)	(8)	(9)	(10)	(11)	(12)	(13)

*f_x_* (μm)	10	5	5	5	5	4	4	4	4	2	2	2	2
*f_y_* (μm)	10	4	3	2	1	4	3	2	1	4	3	2	1
*T* (s)	12	40	50	75	150	64	80	120	240	120	150	225	450

**Figure 2:**
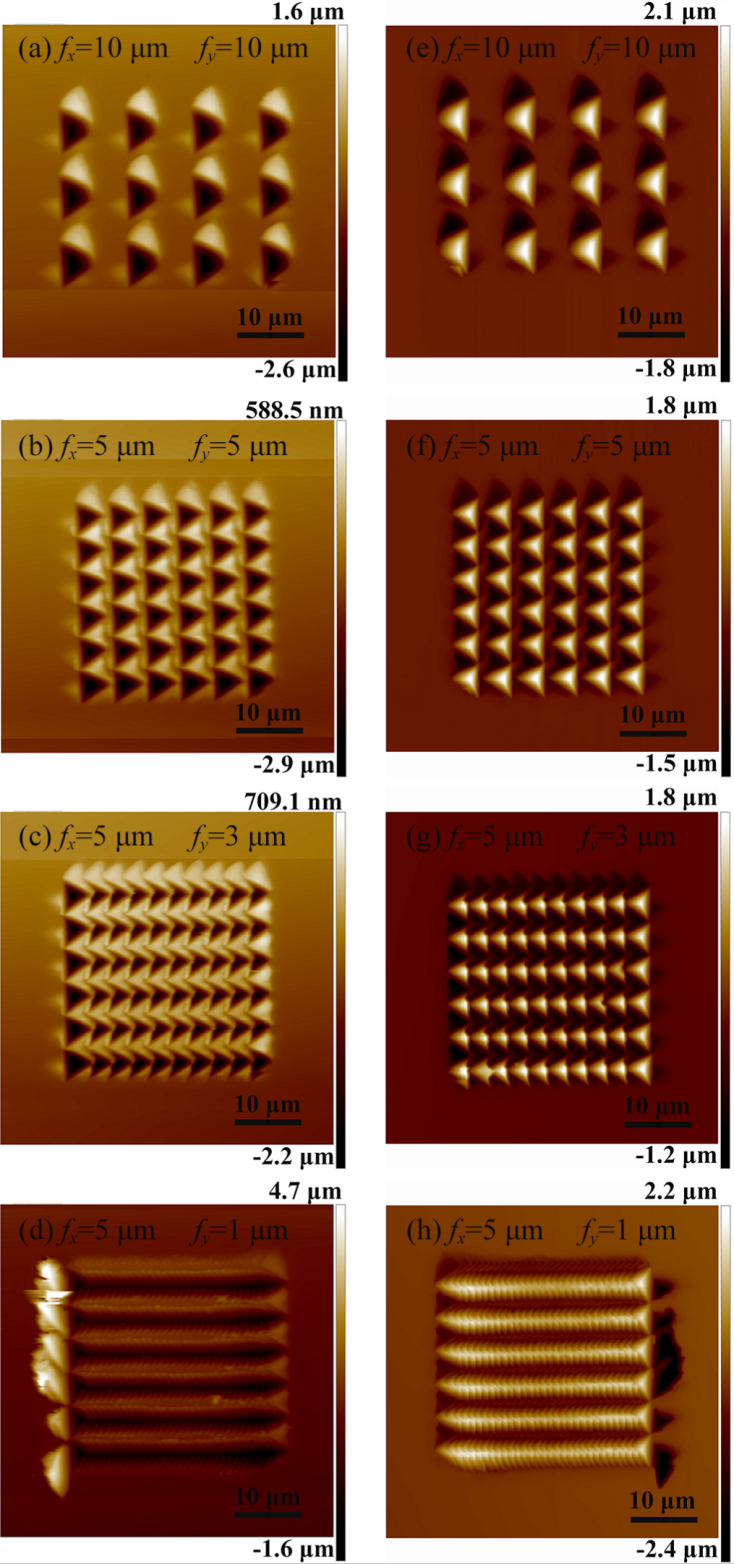
AFM images of cavities with different feed rates (a–d) (with *f_x_* and *f_y_* specified in the images) on a Cu(110) surface. (e–h) Corresponding, replicated micro/nanopyramids on the PDMS surface.

**Figure 3:**
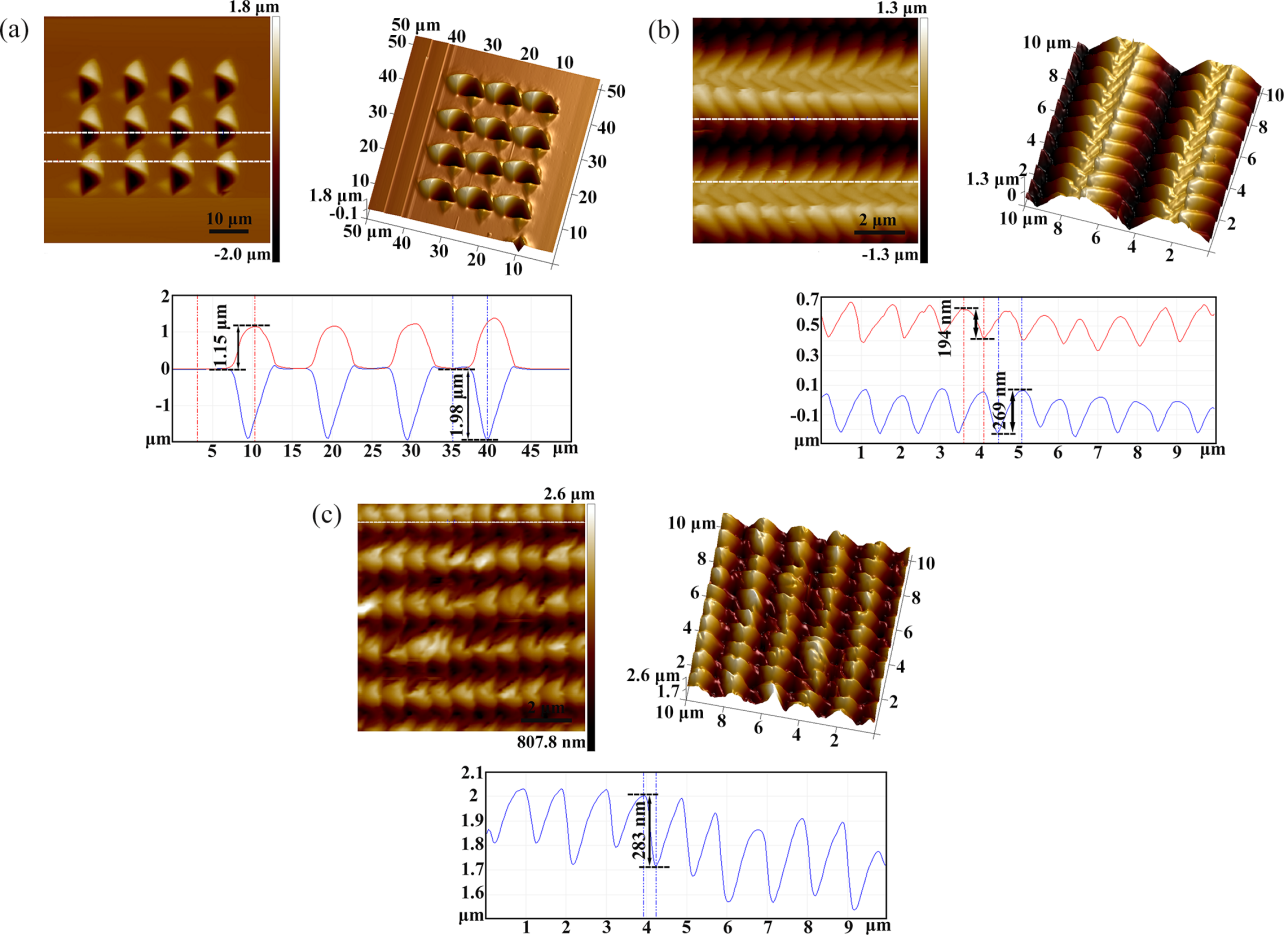
Three-dimensional and sectional AFM images of (a) arrayed pyramids on a Cu(110) surface with *f_x_* = 10 μm and *f_y_* = 10 μm, (b) enlarged arrayed pyramids on a Cu(110) surface with *f_x_* = 5 μm and *f_y_* = 1 μm, and (c) enlarged arrayed pyramids on a Cu (110) surface with *f_x_* = 2 μm and *f_y_* = 1 μm.

Figure 2a–d shows atomic force microscopy (AFM) images of arrayed inverted pyramidal cavities with different feed rates (*f_x_* and *f_y_*) in the *X* and *Y* directions. The adjacent pyramidal inverted cavities were fabricated by moving the material in the *X* and *Z* directions simultaneously, and the *Y* axis was used to obtain two-dimensional microstructures. The feed in the *X* direction ranges from *f_x_* = 5–10 μm and the feed in the *Y* direction ranges from *f_y_* = 1–10 μm. The area of the periodic arrayed cavities is 30 × 30 μm^2^. The amplitude and period of the force signal in the *Z* direction are 10 mN and 1 Hz, respectively. The adjacent pyramidal inverted cavities are separated with feed rates of *f_x_* = 10 μm and *f_y_* = 10 μm using a larger feed step, as shown in Figure 2a. The depth of a single pyramid is 1.98 μm and the height of the pile-up is 1.15 μm, as shown in Figure 3a. When *f_x_* decreased to 5 μm, the pile-up of the adjacent inverted cavities overlapped as shown in Figure 2b. However, when the *f_y_* decreased to 1 μm, the adjacent cavities overlapped and were squeezed in one direction during machining with the decrease of *f_y_*, resulting in the formation of more complex micro/nanostructures on the Cu(110) surface, as shown in Figure 2c,d and Figure 3b,c. Figure 3b shows the three-dimensional and sectional AFM images of arrayed pyramids on the Cu(110) surface with *f_x_* = 5 μm and *f_y_* = 1 μm. The structures of adjacent pyramids were formed with a "fish scale" and the depth of the structure is 269 nm and the height of pile-up is 194 nm. Figure 3c shows the three-dimensional and sectional AFM images of arrayed pyramids on the Cu(110) surface with *f_x_* = 2 μm and *f_y_* = 1 μm. Compared to Figure 3a, smaller arrayed pyramids are formed with *f_x_* = 2 μm and *f_y_* = 1 μm and the depth of adjacent pyramids is 283 nm.

**Figure 4:**
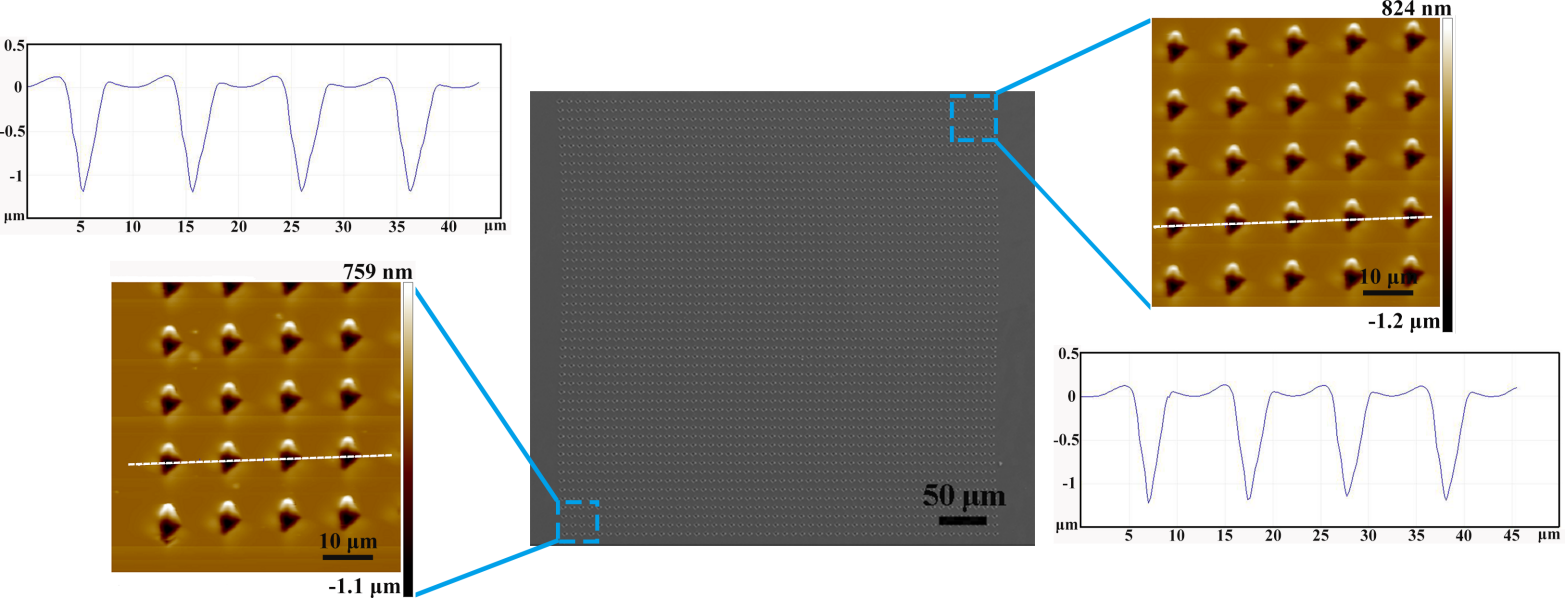
SEM image of the dimension of 500 × 500 μm^2^ arrayed triangular cavities on the Cu (110) surface with *f_x_* = 10 μm and *f_y_* = 10 μm.

Figure 4 shows a scanning electron microscopy (SEM) image of a 500 × 500 μm^2^ array on a Cu(110) surface with *f_x_* = 10 μm and *f_y_* = 10 μm. The sectional AFM images are shown at the machining start stage and end stage, respectively. Using the force modulation method, the depth and width of the cavities are consistent over a large area (500 × 500 μm^2^).

**Figure 5:**
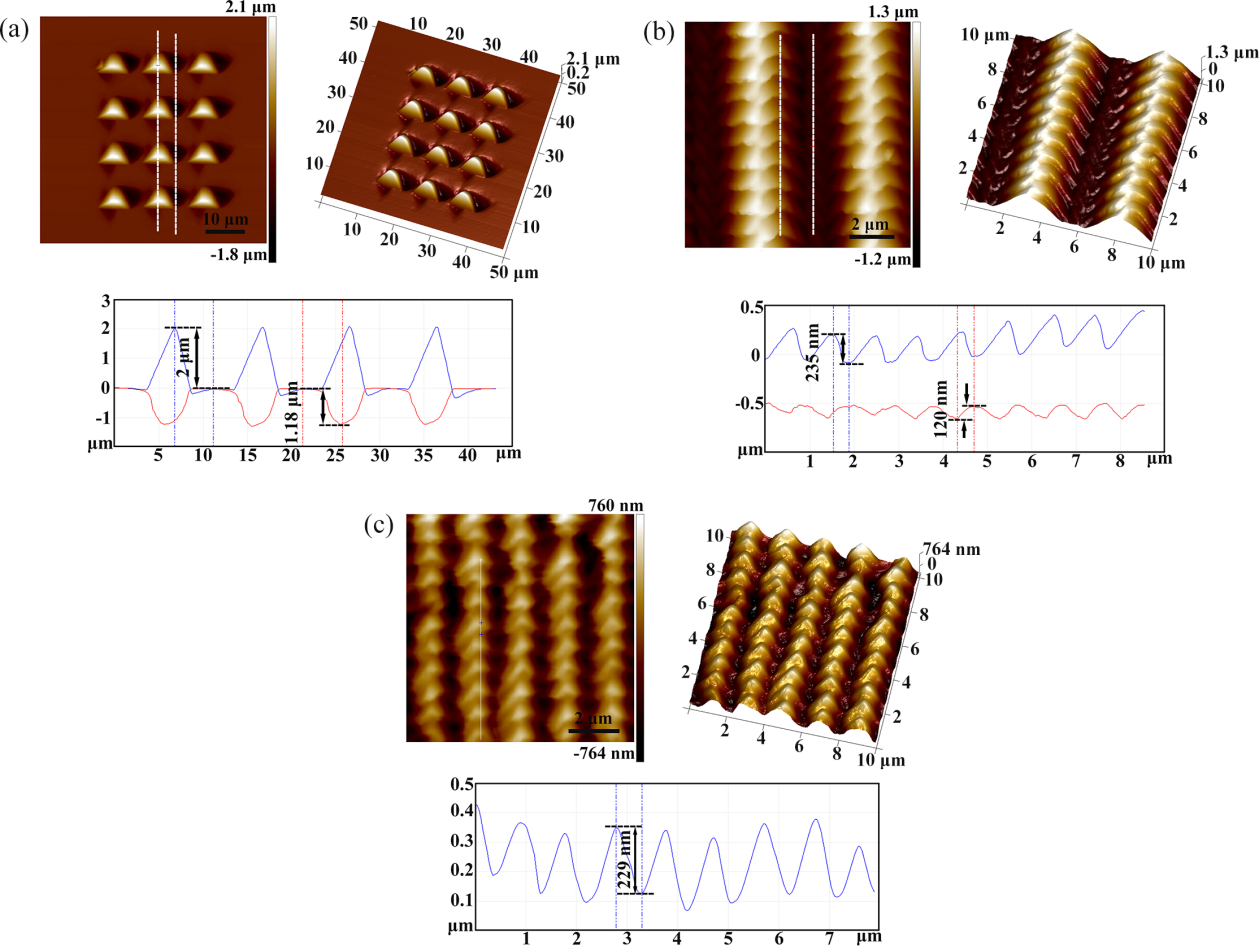
Three-dimensional and sectional AFM images of (a) arrayed pyramids on the PDMS surface after nanoimprinting with *f_x_* = 10 μm and *f_y_* = 10 μm, (b) the enlarged arrayed pyramids on the PDMS surface after nanoimprinting with *f_x_* = 5 μm and *f_y_* = 1 μm for template surface, and (c) enlarged arrayed pyramids on the PDMS surface after nanoimprinting with *f_x_* = 2 μm and *f_y_* = 1 μm.

The hybrid patterns used as the template were then transferred to a PDMS surface using the nanoimprinting and thermal curing processes, and the inverse replicas were successfully achieved. Figure 2e–h shows AFM images of the replicated arrays of micro/nanopyramids on the PDMS surface, corresponding to Figure 2a–d. Figure 5a shows the three-dimensional and sectional AFM images of arrayed pyramids on the PDMS substrate with *f_x_* = 10 μm and *f_y_* = 10 μm; the height of a single pyramid is 2 μm and the depth of the cavity is 1.18 μm. These values are comparable to the original depth of the structure and the height of the pile-up on the Cu(110) surface, as shown in Figure 3a. When the feed (*f_y_*) was reduced to 1 μm, nanostructures of adjacent pyramids with a “fish scale” pattern were also copied onto the PDMS surface after nanoimprinting and the depths of the nanostructures with *f_x_* = 5 μm and *f_y_* = 1 μm were 235 and 120 nm, respectively, as shown in Figure 5b. When the feed rates were decreased in the two directions, smaller pyramids were also generated on the PDMS surface, and the depth of the nanostructures of adjacent pyramids with *f_x_* = 2 μm and *f_y_* = 1 μm is 229 nm, as shown in Figure 5c. Comparing these results with the data in Figure 3 and Figure 5, the depth of the inverted cavities on the Cu(110) surface was basically found to be consistent with the height of the pyramids formed on the PDMS surface. The height of the pile-up on the Cu(110) surface was essentially consistent with the depth of the cavities formed on the PDMS surface as well. Therefore, the structures manufactured by the force modulation indentation method can be copied to a PDMS surface with good homogeneity, and this structured template can be used repeatedly, emphasizing the low-cost nature of this processing procedure.

Finally, the structured PDMS surfaces were coated with a gold film as shown schematically in Figure 1. The gold-coated PDMS substrate was then used as the SERS substrate in the following tests.

### Raman intensity of R6G molecules on structured gold-coated PDMS substrates

In order to study the effect of Raman enhancement of R6G molecules upon excitation on different arrayed pyramidal structures on the gold-coated PDMS surface, the parameters of the feeds (*f_x_* and *f_y_*) for machining the arrayed micro/nanostructures are summarized in Table 1. The Raman mapping data were exported from the Raman spectra point by point. Prior to analysis, first, all Raman spectra were smoothed using a Savitzky–Golay filter with a third-order polynomial and a smooth window size of 13. Second, the baseline of the Raman spectra was adjusted by subtracting a spline interpolation using WiRE 3.4 software.

**Figure 6:**
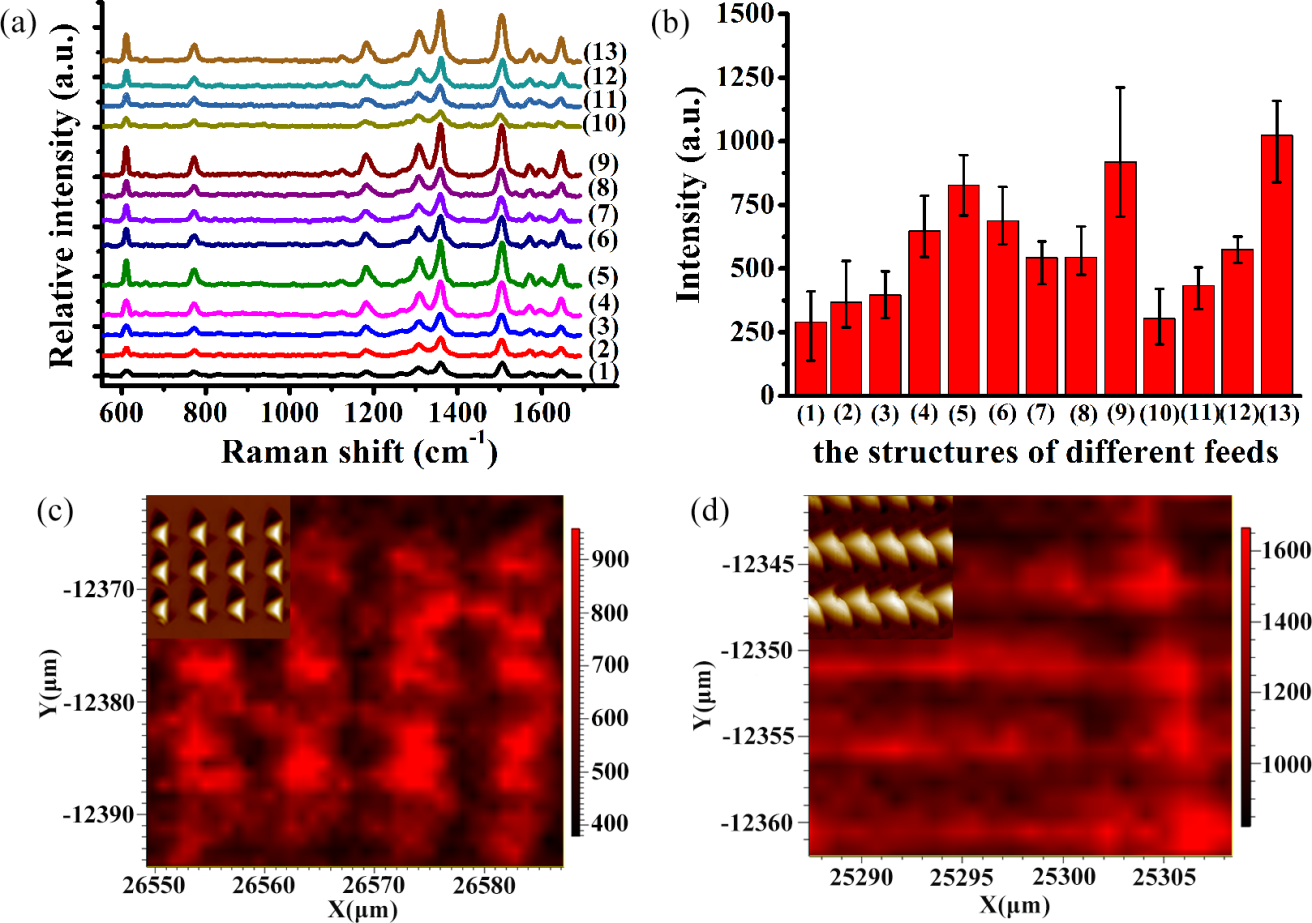
Raman spectra of R6G molecules on a structured PDMS substrate coated with a 10 nm thick gold film at a dye concentration of 10^−6^ M. (a) Raman spectra of R6G molecules on a structured PDMS substrate with different machining structures. Details on the labeling are given in Table 1. (b) Average Raman intensity of the 1362 cm^−1^ R6G peak for a 20 × 20 μm^2^ section of the arrayed pyramid structures with different machining feeds. (c) Raman intensity mapping image of an arrayed PDMS substrate with a feed of 10 μm in the *X* direction and of 10 μm in the *Y* direction and (d) with a feed of 5 μm in the *X* direction and 2 μm in the *Y* direction. The insets are the AFM images corresponding to the ﬁeld map.

Figure 6 shows the Raman spectra of R6G molecules on a PDMS substrate coated with a 10 nm thick gold film with a dye concentration of 10^−6^ M. The characteristic Raman peaks of R6G molecules were successfully identified at 612, 771, 1183, 1311, 1362, 1504 and 1603 cm^−1^, as shown in Figure 6a. Owing to the different microstructures formed by the feeds in the *X* and *Y* directions, it can be observed that the Raman enhancement can be remarkably affected by these structures.

**Figure 7:**
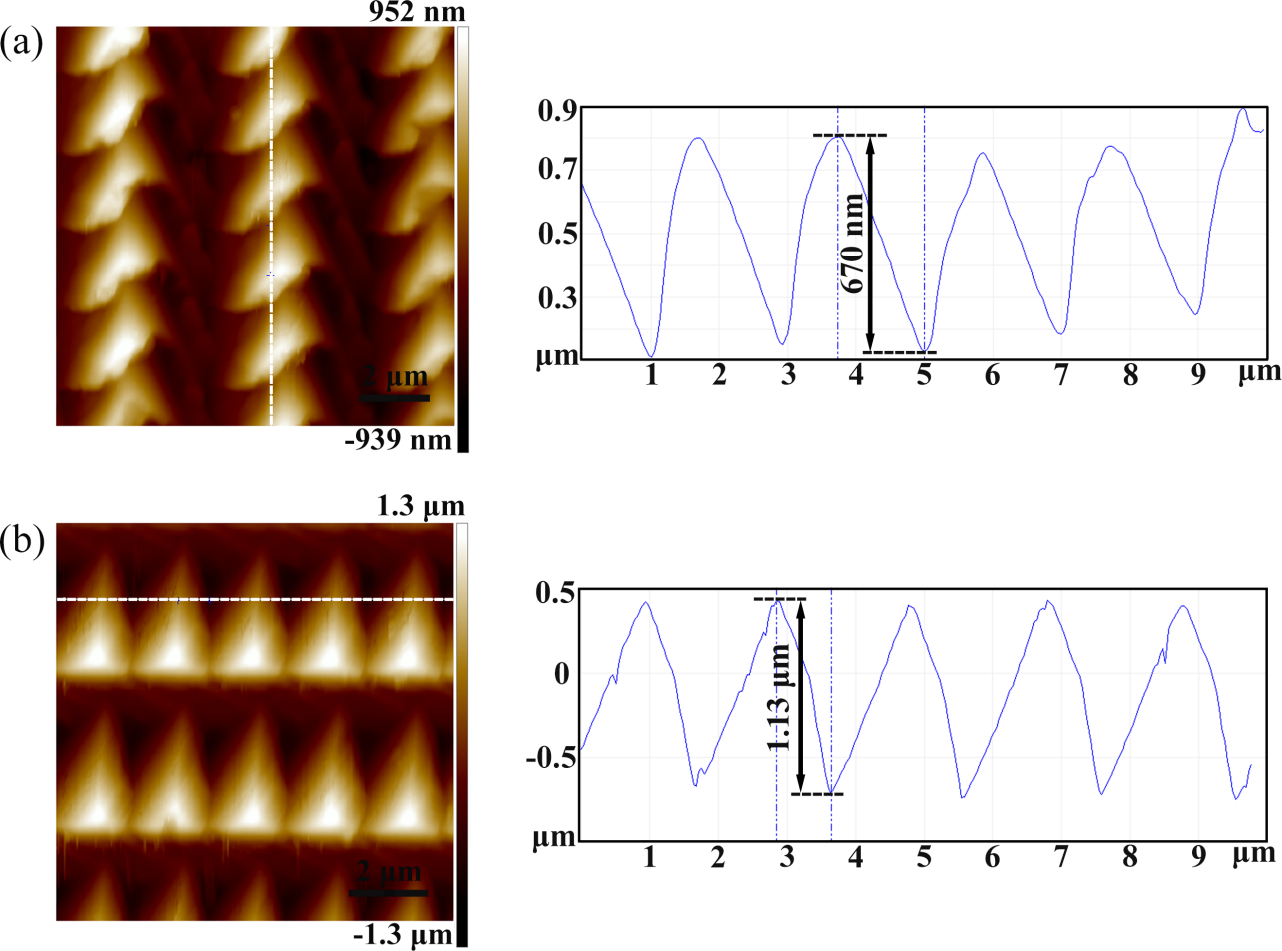
Three-dimensional and sectional AFM images of (a) arrayed pyramids on the PDMS surface after nanoimprinting with *f_x_* = 4 μm and *f_y_* = 2 μm and (b) arrayed pyramids on the PDMS surface after nanoimprinting with *f_x_* = 2 μm and *f_y_* = 4 μm for the template surface.

All the characteristic peaks of the Raman intensity in curve (1) in Figure 6a have a weaker enhancement, corresponding to Figure 2e and Figure 5a. Under this condition, the adjacent pyramids do not overlapped with each other. When the feed (*f_x_*) is set to 5 μm in the *X* direction, the Raman intensity of the 1362 cm^−1^ peak of R6G increases with decreasing *f_y_*, as can be seen in the curves (2)–(5). The Raman intensity for the case of *f_y_* = 1 μm is the strongest, as can be seen in all curves shown in Figure 6a. Figure 6b shows the average Raman intensity of the 1362 cm^−1^ R6G peak of a 20 × 20 μm^2^ area of the arrayed pyramids with different feeds, corresponding to Figure 6a. Owing to the different machining parameters (*f_x_* and *f_y_*), a variety of pyramidal structures are formed on the PDMS substrates. The Raman intensity for the case of *f_y_* = 1 μm in the *Y* direction is the strongest, corresponding to lines (5), (9) and (13) in Figure 6b. In this case, the Raman intensity of the 1362 cm^−1^ R6G peak does not significantly change for the other machining parameters. For example, the height of the structures produced with feed rates of *f_x_* = 2 μm and *f_y_* = 4 μm is 1.13 μm and the height of those with *f_x_* = 4 μm and *f_y_* = 2 μm is 670 nm, as shown in Figure 7. This is because the length of a triangle (*X* direction) is longer than the height of a triangle (*Y* direction) for the projection area of a single cavity. The height of structures produced with feed rates of *f_x_* = 4 μm and *f_y_* = 2 μm is shallower than that of structures with *f_x_* = 2 μm and *f_y_* = 4 μm. Therefore, compared to the 2 μm feed in the *X* direction and 4 μm in the *Y* direction, the Raman intensity for *f_x_* = 4 μm and *f_y_* = 2 μm is higher, as shown in Figure 6b.

The strongest Raman intensities of the 1362 cm^−1^ R6G peak were detected by two different structures, as shown in Figure 5b,c. The main reason for this enhancement is due to the micro/nanostructures formed using different feeds and the shallower depth of the nanostructures which results in the variation in the Raman intensity of the probe molecules. The adjacent pyramids begin to overlap with a decrease of the feed and more nanostructures are generated in this case. When the feed is decreased in one direction, a “fish scale” type of nanostructured pattern is generated, as shown in Figure 5b. When the velocities are decreased both directions, smaller pyramids are formed, as shown in Figure 5c. The height of the structures and the space between adjacent pyramids gradually decreases with decreasing the feed. Therefore, the area density of the structures increases gradually, as shown in Figure 5. The Raman intensity is enhanced with the smaller feed rates under the same detection conditions, as shown in Figure 6a,b. Figure 6c,d shows Raman intensity mapping images of the arrayed PDMS substrate with a feed of 10 μm in the *X* direction and of 10 μm in the *Y* direction and a feed of 5 μm in the *X* direction and of 2 μm in the *Y* direction, respectively. The inset is an AFM image corresponding to the ﬁeld map. It has been experimentally verified that the Raman intensity distribution is uniform along the same direction. Combining the force modulation indentation method with the nanoimprinting method, it is shown that the flexible, transparent PDMS substrates coated with gold are highly reproducibility and long-term stable. Researchers have already previously obtained similar results of the effects of nanostructures on the SERS measurement. First, some researchers [18–19] have studied the phenomenon in which the probe molecules become trapped within the nanostructures, which is easily detectable by the SERS effect. Therefore, it can be deduced that more R6G molecules can be adsorbed and entrapped by the nanostructures. Second, it is known that the electromagnetic mechanism (EM) with a strong local electromagnetic field is the main factor which induces the Raman enhancement for different micro/nanostructures. Compared to a bare surface, the enhanced electric field can be generated by a single pyramid. In addition, the apex of a single pyramid can induce a significant enhancement and an enhanced Raman signal with respect to the other parts of the pyramid [4,35–36]. Moreover, researchers [37–38] have verified that the stronger enhancement of the electromagnetic field happens at the locations between adjacent pyramids. This indicates that the nanostructures formed by adjacent pyramids play a critical role in determining the electric field amplitude distribution and the corresponding Raman enhancement factor. The electric field intensity generated by adjacent structures is higher than the electric field intensity generated by dispersed structures [37]. Therefore, compared to the dispersed pyramids, the uniformity and stability of the electric field intensity from the molecules adsorbed on the numerous hot spots formed by adjacent pyramids would be significantly enhanced, which agrees well with our experimental results.

**Figure 8:**
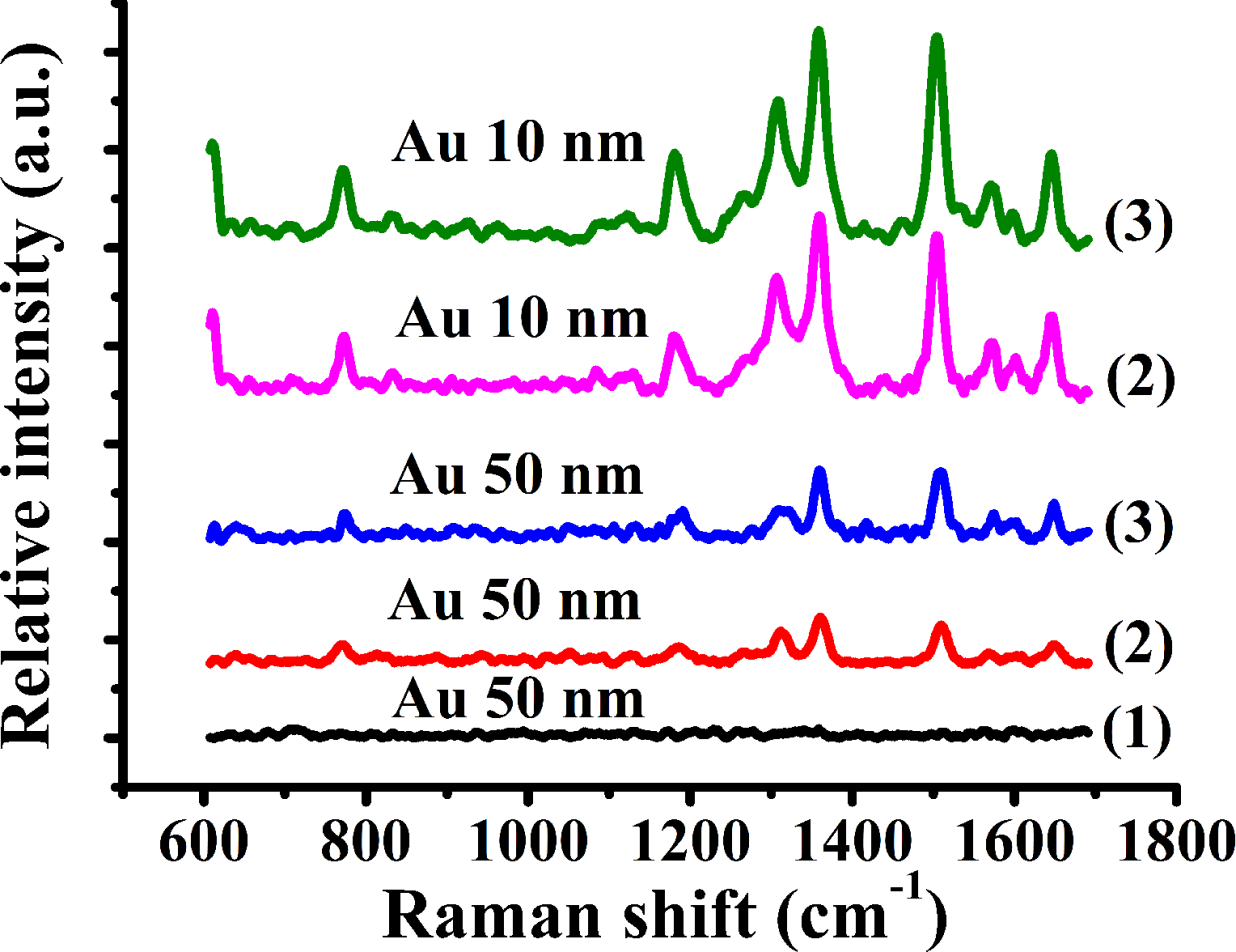
Raman spectra of R6G molecules on the gold-coated PDMS substrate at a concentration of 10^−6^ M with different gold film thicknesses and different feed rates.

**Figure 9:**
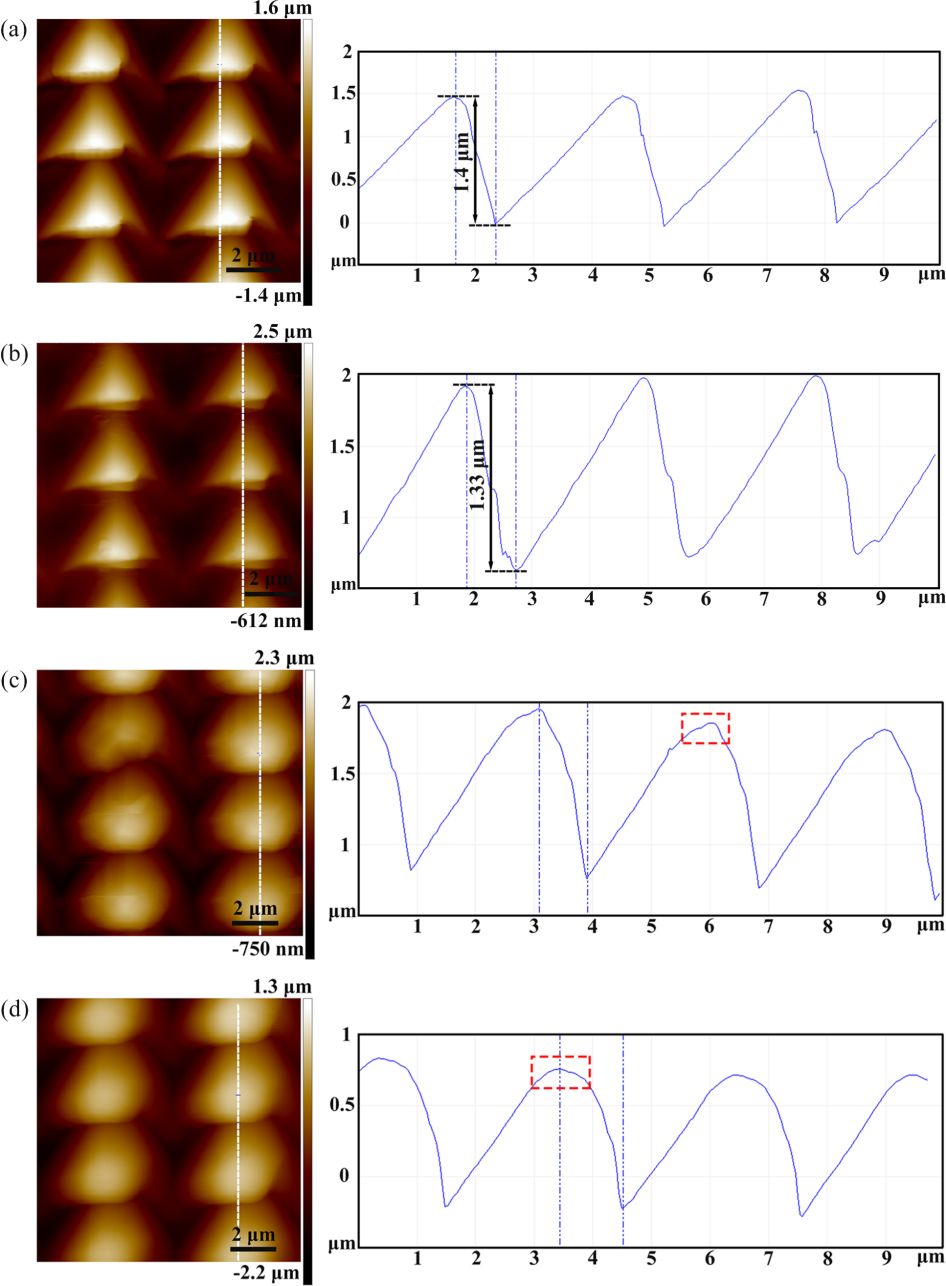
Two-dimensional and sectional AFM images of arrayed pyramids on PDMS substrates (a) the first time the template was used with *f_x_* = 5 μm and *f_y_* = 3 μm, (b) the fourth time the template was reused with *f_x_* = 5 μm and *f_y_* = 3 μm, (c) with a 10 nm thick gold film on the PDMS substrate with *f_x_* = 5 μm and *f_y_* = 3 μm, and (d) with a 50 nm thick gold film on the PDMS substrate with *f_x_* = 5 μm and *f_y_* = 3 μm.

Figure 8 shows the Raman spectra of R6G molecules on the gold-coated PDMS substrate at a concentration of 10^−6^ M and with different thicknesses of the gold film and different feed rates (*f_x_* and *f_y_*). The thicknesses of the gold films were 10 nm and 50 nm, respectively, and the the *f_x_* and *f_y_* feed rates were chosen using the parameters for (2) and (3) in Table 1. Figure 9 shows two-dimensional and sectional AFM images of the arrayed pyramids with different gold film thicknesses on PDMS substrates with *f_x_* = 5 μm and *f_y_* = 3 μm. The height of the structures is 1.4 μm after the first time the template was used, as shown in Figure 9a, and a height of 1.33 μm was measured after the fourth time the template was used, as shown in Figure 9b. The dimensions and shape of the structures did not significantly change with increased use of the template. The top of the pyramid was gradually flattened with increasing thickness of the gold film. The Raman intensity of R6G with a 10 nm thick gold film was stronger than that with the same PDMS substrate coated with a 50 nm thick gold film. However, we could not detect any R6G signal with the 50 nm thick gold film on the PDMS pyramidal substrate when the feed of 10 μm (both in the *X* and *Y* directions) was used, as shown in Figure 8. The influence of gold thickness on the electric field intensity has been previously studied [39–40]. Gao et al. [39] investigated nanostructures coated with approximately 15 nm of Au that exhibited a very strong SERS effect. The thinner the Au films, the higher the Raman intensity signal. Vernon et al. [40] determined that the hot spot signal is approximately constant for gold film thicknesses greater than 125 nm. Additionally, the Raman intensity for a gold film thickness of 75 nm was found to be higher than that of a standard Klarite pit coated with a 300 nm thick gold film. Therefore, it can be concluded that the Raman intensity of probe molecules can be enhanced by employing a thinner gold film.

**Figure 10:**
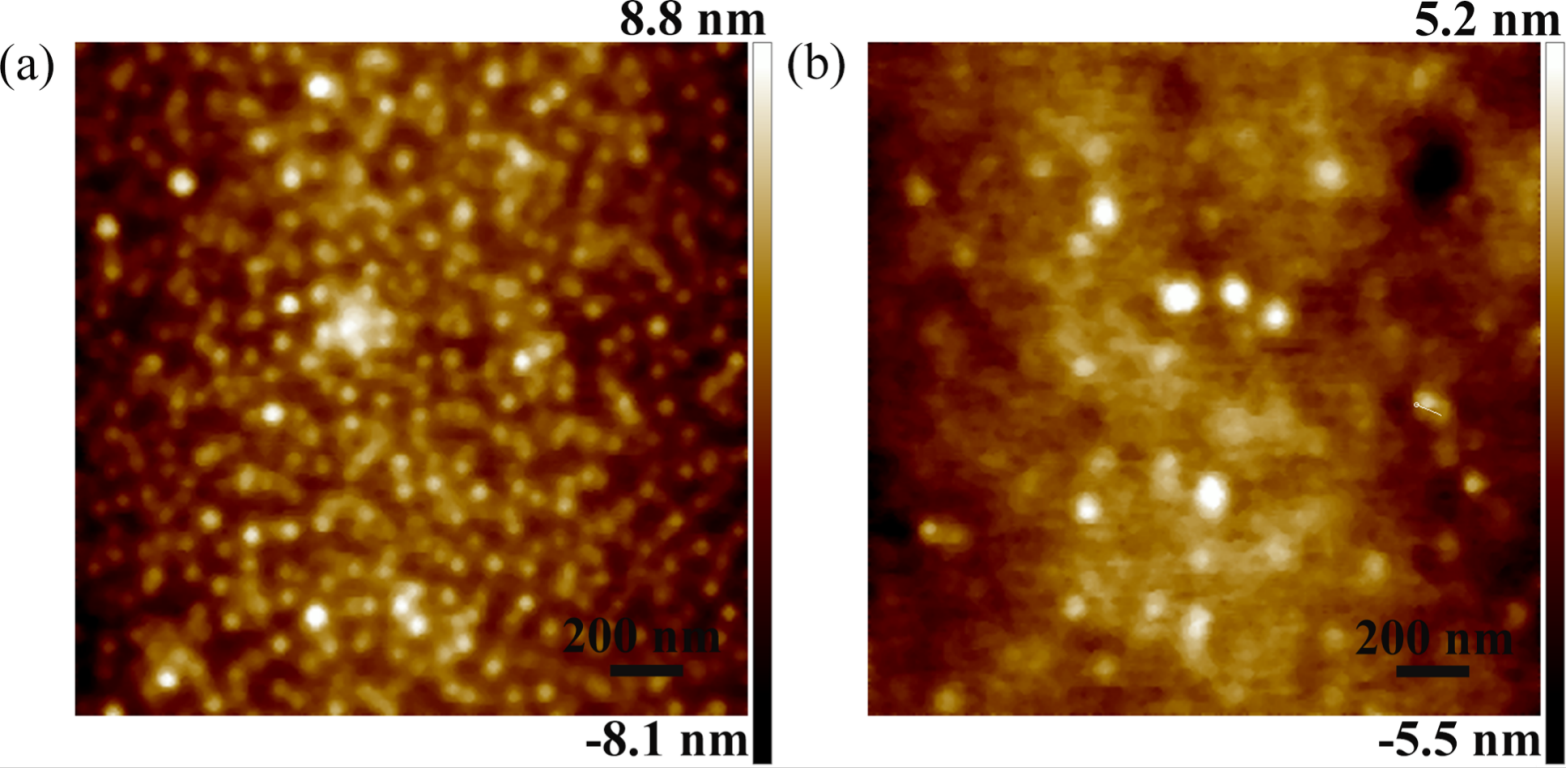
AFM images of the gold films of (a) 10 nm (b) 50 nm thickness on PDMS substrates.

Figure 10 shows AFM images of PDMS substrates coated 10 and 50 nm thick gold films. Compared to the gold film thickness of 50 nm, more gold nanoparticles are generated on the PDMS surface with a gold film thickness of 10 nm, as shown in Figure 10a,b. The average size of the nanoparticles for a gold-film thickness of 10 and 50 nm is approximately 45 and 90 nm, respectively. The roughness of the PDMS surface with a gold film thickness of 10 and 50 nm is 1.9 and 1.2 nm, respectively, under the same measurement parameters. More hot spots were detected with the increasing quantity of nanoparticles for the dense surface with a gold film thickness of 10 nm. Similar results were obtained in previous research [41–42], and it was found that even a single nanoparticle can serve as a hot spot to enhance the Raman intensity. In addition, the local EM field can be enhanced further by reducing the gap between adjacent nanoparticles and the largest enhancement was observed with a dense surface of nanoparticles.

**Figure 11:**
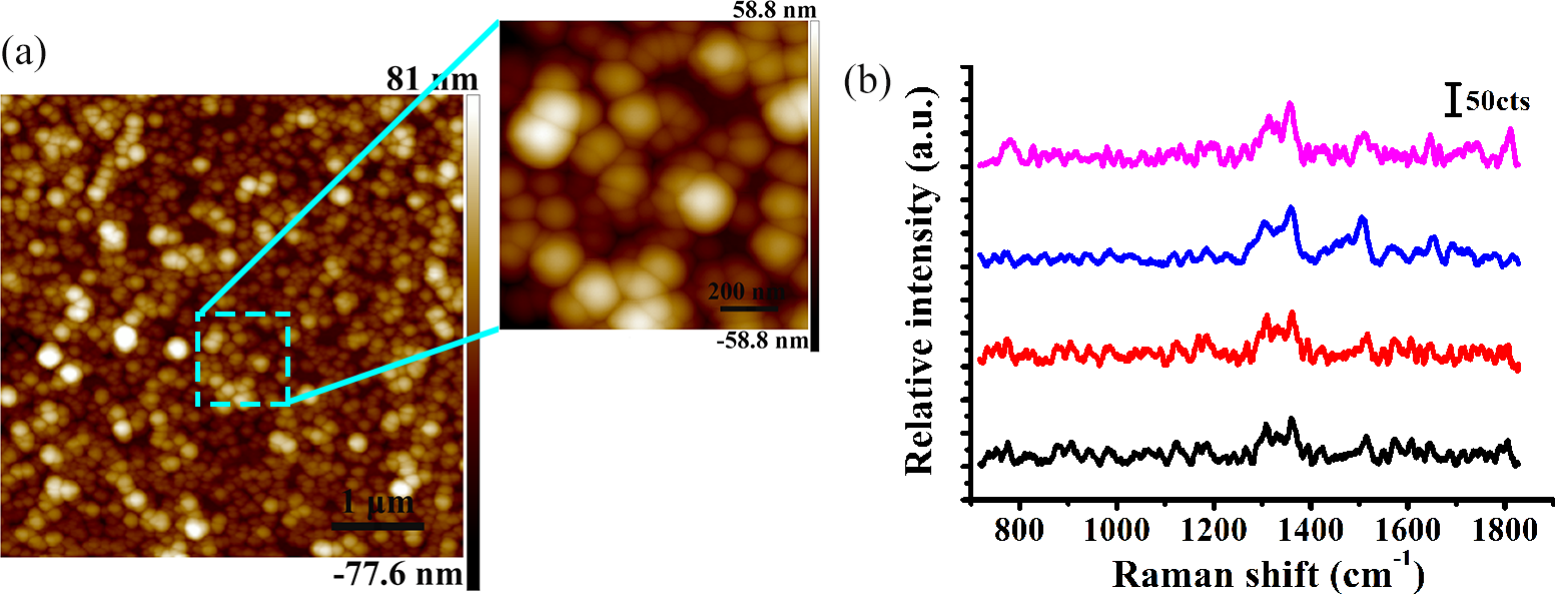
(a) AFM image of the commercial Q-SERS substrate and an enlarged image of the local position. (b) Raman spectra of R6G molecules at a concentration of 10^−6^ M on the commercial Q-SERS substrate at different locations.

Figure 11 shows an AFM image of the commercial Q-SERS substrate (gold nanoparticles coated on a 5 × 5 mm^2^ silicon wafer) with an enlarged image of the local position and the Raman spectra of R6G molecules at a concentration of 10^−6^ M on the commercial Q-SERS substrate. The nanoparticle diameter was found to be approximately 150 nm and the roughness of the Q-SERS substrate was 9.5 nm. The Raman intensity of the 1362 cm^−1^ R6G peak is approximately 110 counts on the Q-SERS substrate at different positions, as shown in Figure 11b. Because the Q-SERS substrate is characterized by the deposition of Au nanoparticles on a flat silicon surface, the enhancement of Au nanoparticles was detected only on the commercial Q-SERS substrate, as shown in Figure 11. However, the pyramidal structures were fabricated using different parameters by employing the proposed method and deposition of Au nanoparticles. The enhancement of these two factors was thus achieved. Therefore, the intensity when using the Q-SERS substrate can be considered to be relatively low. In addition, the average Raman intensity of the 1362 cm^−1^ R6G peak ranges from 250 to 1050 counts on the gold-coated PDMS substrate under the same detection conditions in the experiments, as shown in Figure 6b. Therefore, compared to a commercial Q-SERS substrate, the pyramidal structures machined in the present study exhibit a stronger enhancement.

The enhancement factor of the structured gold-coated PDMS surface can be calculated as follows [43–44]:

[1]
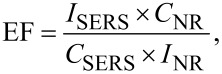


where *I*_SERS_ and *I*_NR_ are the intensities of SERS and normal Raman scattering, respectively; *C*_SERS_ and *C*_NR_ are the concentration of the dye molecule in the SERS and normal Raman measurements, in this case using a 0.25 mol R6G solution on a SiO_2_ wafer. The SERS enhancement factor for the various micro/nanostructures is between 7.5 × 10^5^ and 6 × 10^6^ for the structured PDMS substrate formed by the method described in the present study.

## Conclusion

Two- and three-dimensional arrayed micro/nanopyramids were fabricated on PDMS substrates by combining the tip-based force modulation indentation method with the reverse nanoimprinting process. By optimizing the machining parameters for the template, such as the feed rate, the complex, arrayed micropyramids along with the nanostructures were achieved based on the overlap and extrusion of the adjacent micropyramids. The SERS intensity of R6G molecules was verified and improved further by coating the structured PDMS surface with a thin gold film. In addition, the Raman intensity of the 1362 cm^−1^ R6G peak with adjacent pyramidal structures was stronger than that of the same peak with a commercial Q-SERS substrate. The SERS enhancement factor of the gold-coated, structured PDMS substrate was between 7.5 × 10^5^ and 6 × 10^6^ , which demonstrates that this proposed method is a reliable, replicable, homogeneous, low cost process, and offers high Raman enhancement ability. Combining the force modulation indentation with the reverse nanoimprinting method, the structured SERS substrates with a flexible, transparent, gold-coated PDMS substrate can be used not only to detect liquid solutions, but also irregular surfaces, such as pesticide residues on the skin of fruit or fish.

## Experimental

To perform the Raman measurements, the structured PDMS samples were coated with a Au film and dipped into a R6G aqueous solution with a concentration of 10^−6^ M for 30 min. They were then rinsed with ethanol to remove the excess R6G molecules and dried with a continuous, gentle nitrogen flow. A micro-Raman spectroscopic system (Renishaw, inVia, UK) equipped with a 633 nm wavelength laser and focused with a 50× objective lens was employed. The incident optical power was 0.6 mW and the beam diameter was approximately 1 μm. The signal detector used a Renishaw CCD camera (1040 × 256) a grating size of 1800 lines/mm was employed. The exposure time was set to 1 s and one accumulation scan was made. The mapping images of the micro-Raman spectrum were scanned over a 20 × 20 μm^2^ area. Before the tests, a standard Si substrate was employed to rectify the Raman spectrum, and no specific peaks were found. The Raman intensity R6G probe peak was chosen as 1362 cm^−1^ for the experiment, which is the major Raman peak for R6G molecules.

A Dimension Icon AFM system (Bruker, Germany) was used to observe the topography of the machined micro/nanostructures. The scan size was 50 × 50 μm^2^. The elastic constant of the silicon cantilever was 0.2 N/m and contact mode was employed. A Merlin Compact SEM system (Zeiss, Germany) was employed to detect the machined structures on a large scale.
